# Supplementary material for: Phase synchronization of fluid-fluid interfaces as hydrodynamically coupled oscillators
Source: Nat Commun. 2020 Oct 15;11:5221. doi: 10.1038/s41467-020-18930-7 (PMC7562928; doi:10.1038/s41467-020-18930-7)
Supplement: Supplementary file 1 — Supplementary Information [file 41467_2020_18930_MOESM1_ESM.pdf]

Supplementary Information

Phase synchronization of fluid-fluid interfaces as hydrodynamically coupled oscillators

Eujin Um,[1,*] Minjun Kim,[1] Hyoungsoo Kim,[2] Joo H. Kang,[3] Howard A. Stone,[4] Joonwoo Jeong[1,*]

[1] Department of Physics, Ulsan National Institute of Science and Technology (UNIST), Ulsan, 44919, Republic of Korea.

[2] Department of Mechanical Engineering, Korea Advanced Institute of Science and Technology (KAIST), Daejeon, 34141, Republic of Korea.

[3] Department of Biomedical Engineering, Ulsan National Institute of Science and Technology (UNIST), Ulsan, 44919, Republic of Korea.

[4] Department of Mechanical and Aerospace Engineering, Princeton University, Princeton, New Jersey 08544, USA.

*Corresponding authors: E. U. (eujinum@unist.ac.kr) and J. J. (jjeong@unist.ac.kr)

Supplementary Figures

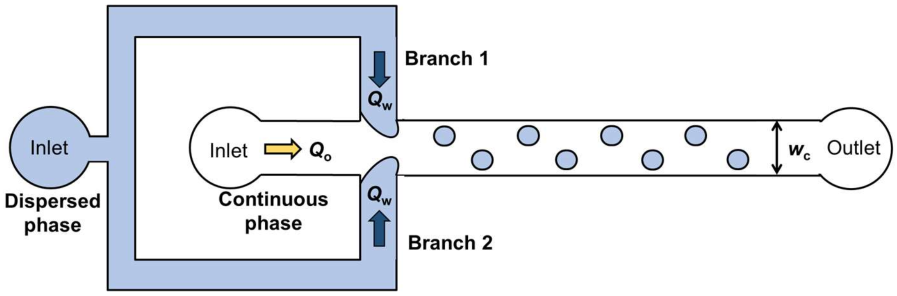

Supplementary Fig. 1 Sketch of double T-junction microchannel with two interfaces. The continuous phase (oil) flows through the main channel of width w_c, while the dispersed phase (water) flows through two opposing side branches to form two interfaces at the double T-junction. Q_o and Q_w indicate the volumetric flow rates of the oil and water phases, respectively.

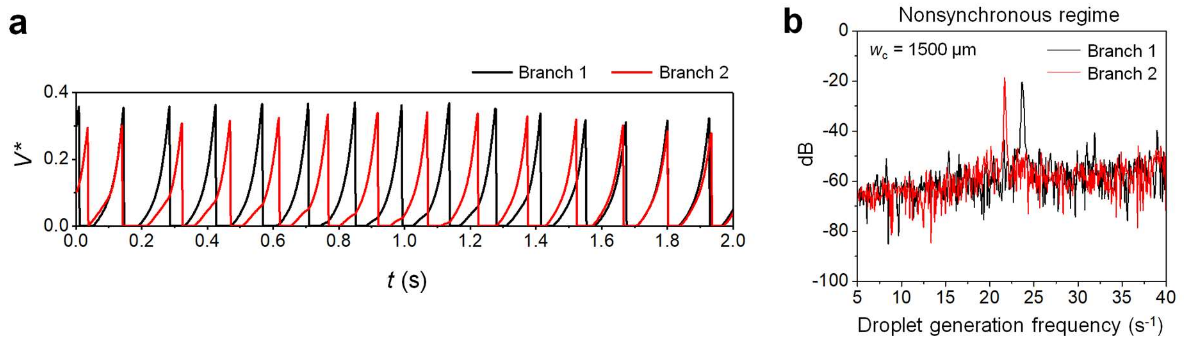

Supplementary Fig. 2 Nonsynchronous regime of droplet generation. (**a**) The change in the volume of the dispersed phase of each branch with respect to time at w_c = 1500 μm (Q_o = 1500 μL h^{-1} and Q_w = 100 μL h^{-1}), showing independent generation of droplets. The volume, V^*, is scaled by the volume of the junction $w_c w_d h$, where w_c, w_d, h are the widths of the main channel and branch, and the height of the channel, respectively. (**b**) Power spectra derived from the Fourier transform (FFT) of experimentally measured time series of droplet breakup. A sequence of at least 500 droplets was analyzed.

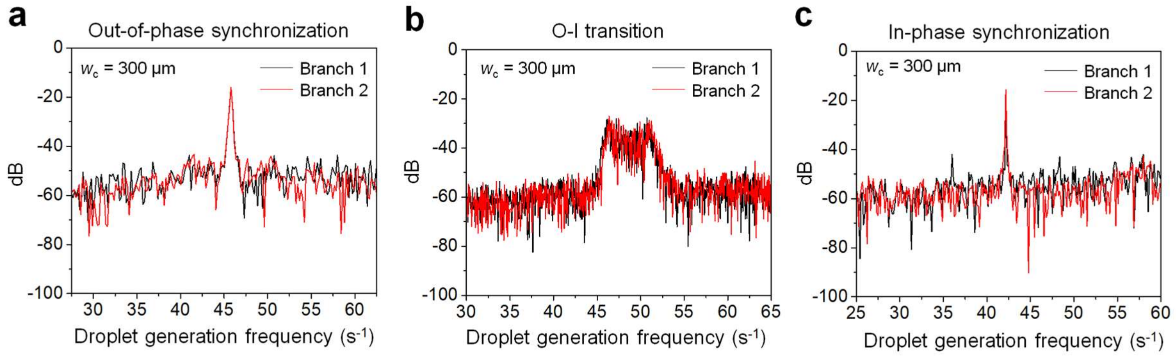

Supplementary Fig. 3 Droplet generation frequencies at each synchronous regime. Power spectra derived from the Fourier transform (FFT) of experimentally measured time series of droplet breakup at w_c = 300 μm for the (**a**) out-of-phase synchronization, (**b**) transition between the out-of-phase and in-phase (O-I transition) mode, and (**c**) in-phase synchronization mode. The flow rate conditions used to obtain this data were the same as those given in Fig. 1 in the main text. A total of at least 500 droplets was analyzed in each condition.

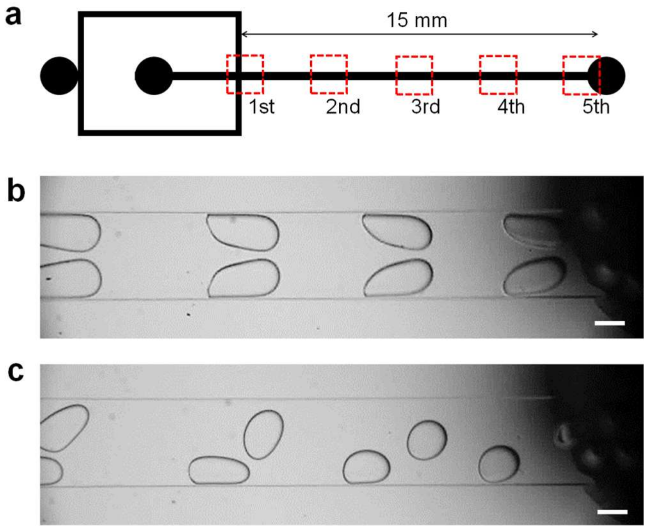

Supplementary Fig. 4 Observation of droplet pairs along the channel in in-phase mode. (**a**) Five regions along the main channel towards the outlet are observed on the microscope as shown in Supplementary Movie 5. (**b**) The image captured at the 5th region of the channel showing that the droplet pairs maintain the side-by-side configuration until the end of the channel, when Q_o = 300 μL h^{-1}, Q_w = 50 μL h^{-1}. The dark region on the right end of the image is the outlet hole, and the shape of droplets seems to change towards the outlet due to the distortion in the outlet region caused by the insertion of the outlet tubing. (**c**) The side-by-side configuration could be disrupted towards the outlet if the size of droplets become smaller, when Q_o = 300 μL h^{-1}, Q_w = 25 μL h^{-1}. Scale bars = 100 μm.

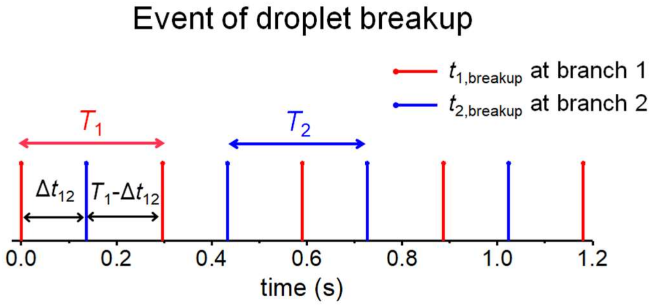

The state of droplet generation ($T_1 \cong T_2$)	$\alpha = \frac{\max(\Delta t_{12}, T_1 - \Delta t_{12})}{T_1}$
In-phase synchronization	$0.8 < \alpha \leq 1$
Out-of-phase synchronization	$0.5 \leq \alpha \leq 0.8$
O-I Transition or nonsynchronous regime	N.A.

Supplementary Fig. 5. Schematic graph showing timestamps of the droplet breakup. By obtaining the time stamp of the droplet breakup from each branch, $t_{1,breakup}$ (red), and $t_{2,breakup}$ (blue), we can define the synchronization parameter α, which establishes criteria for categorizing the droplet-generating mode: in-phase or out-of-phase synchronization, and transition between the out-of-phase and in-phase (O-I transition) regime or nonsynchronized regime. The period of droplet breakup from branch 1 is T_1, and from branch 2 is T_2, and the time difference between the droplet breakup in branches 1 and 2 is Δt_{12} and $T_1 - \Delta t_{12}$, respectively. α = 1 indicates perfect in-phase synchronization.

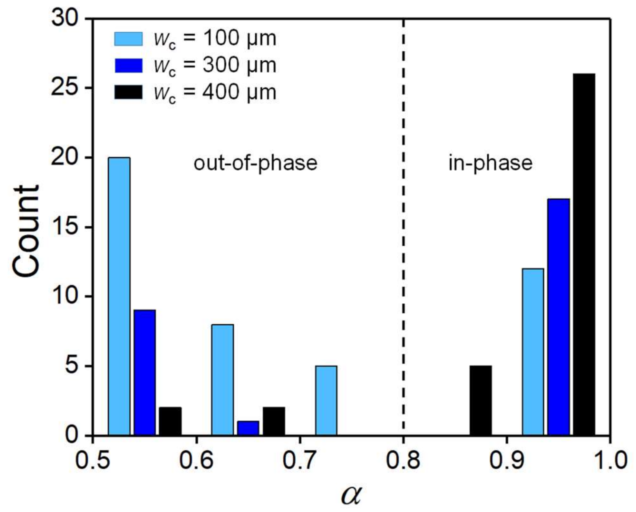

Supplementary Fig. 6 Histogram showing the distribution of the synchronization parameter, *α*. The distribution of *α* in in-phase or out-of-phase synchronization is obtained from the experimental results with the microchannels of various w_c. The dashed line corresponds to the criterion, $\alpha = 0.8$, which discriminates the in-phase and out-of-phase synchronization modes.

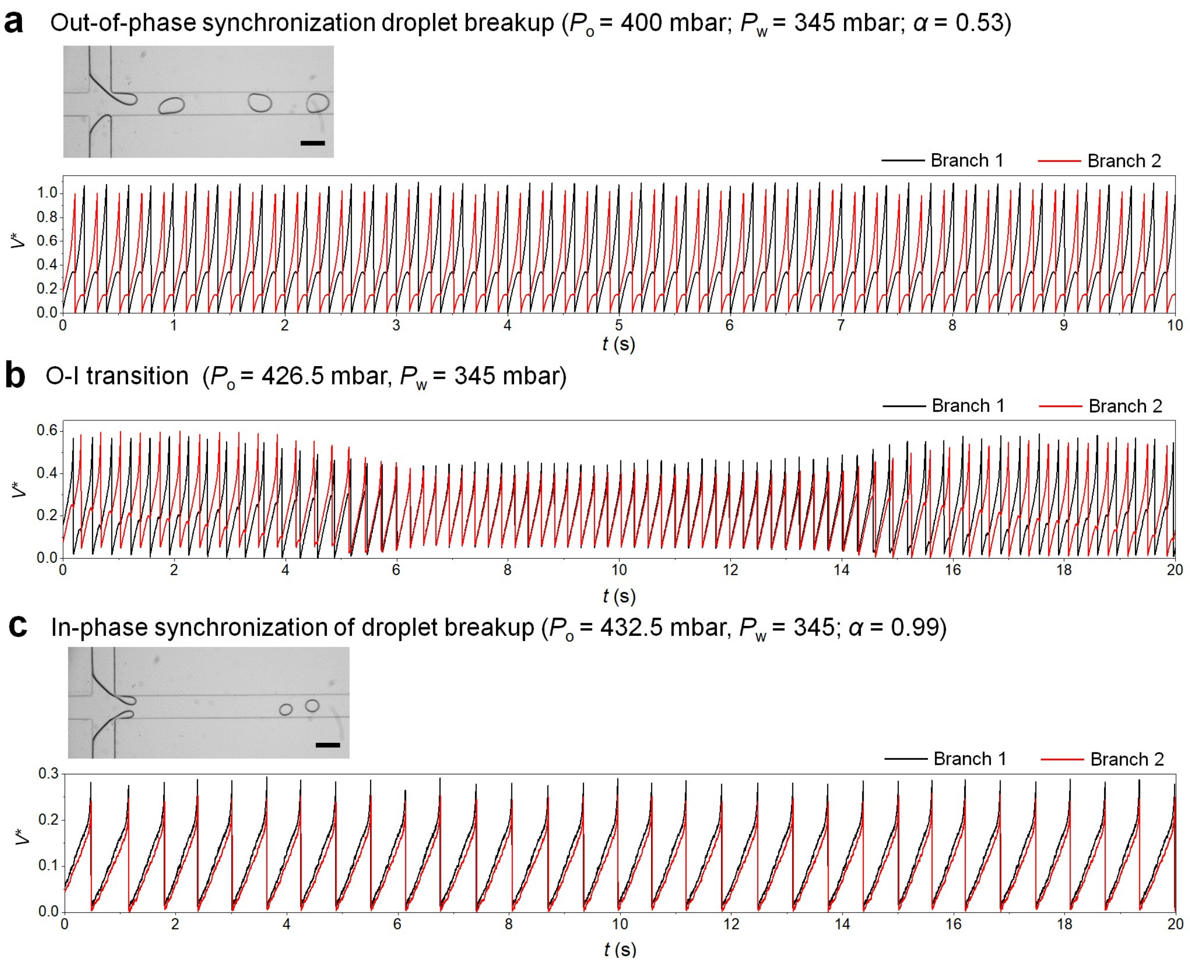

Supplementary Fig. 7 Results of the synchronization modes using pressure pumps. Pressure pumps were used in the experiments to apply constant pressures to the continuous phase (P_o), and dispersed phases (P_w), at the inlet without the pulsing effect from the syringe pumps (w_c = 100 μm). Three modes of droplet breakup at the double T-junction, (**a**) out-of-phase, (**b**) transition between the out-of-phase and in-phase (O-I transition), and (**c**) in-phase synchronization modes, were observed, as shown by plots of the scaled volume, V^*, of the protruded dispersed phase from each branch versus time. The volume, V^*, is scaled by the volume of the junction $w_c w_d h$, where w_c, w_d, h are the widths of the main channel and branch, and the height of the channel, respectively. As the pressure $P_w P_o^{-1}$ ratio decreases, the droplet generation mode changes from the out-of-phase to the in-phase mode, via the O-I transition. All scale bars indicate 100 μm.

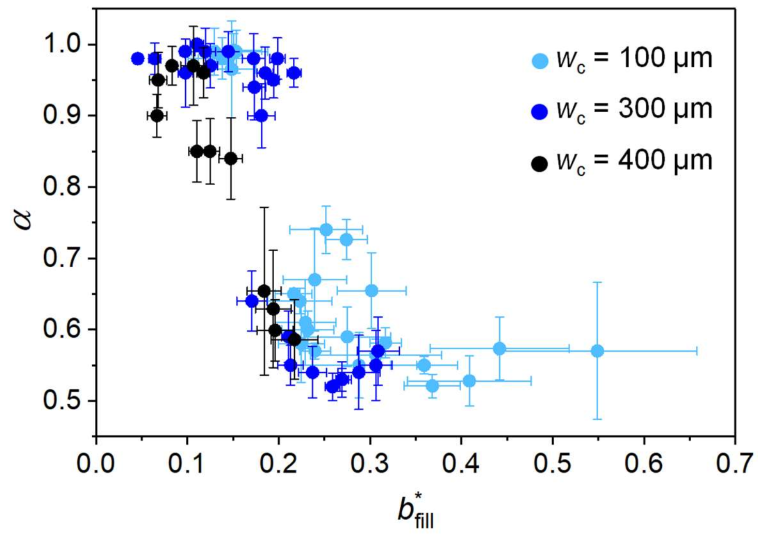

Supplementary Fig. 8 Plot of the synchronization parameter, *α*, versus b^*_{fill}. Each data point is the average value of *α* and the normalized protrusion length, $b^*_{\mathrm{fill}} = b_{\mathrm{fill}} w_c^{-1}$ measured from an image sequence containing, at least, 500 droplets; The vertical and horizontal error bars indicate standard deviations.

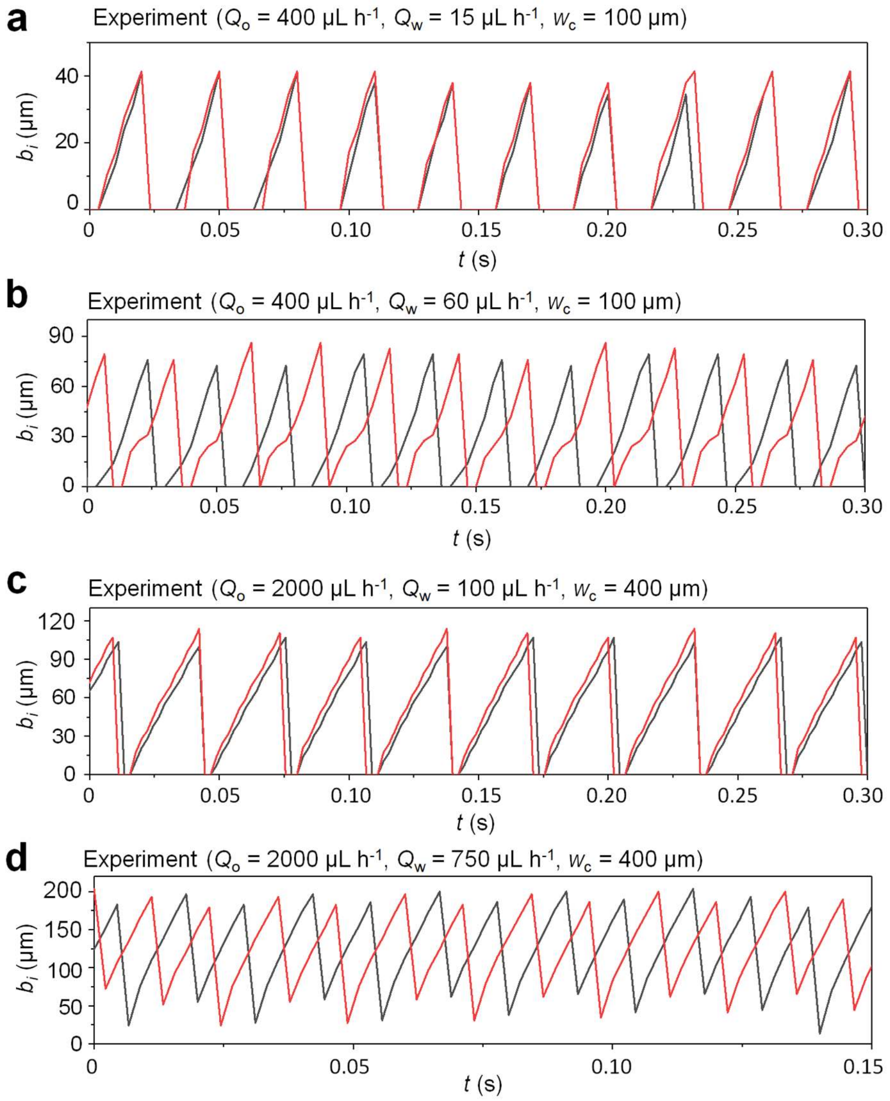

Supplementary Fig. 9 Plots of protrusion height *b* as a function of time *t*. The protrusion height of each branch was measured at various experimental conditions. (**a**) In-phase synchronization and (**b**) out-of-phase synchronization mode for w_c = 100 μm, and (**c**) in-phase and (**d**) out-of-phase synchronization mode for w_c = 400 μm.

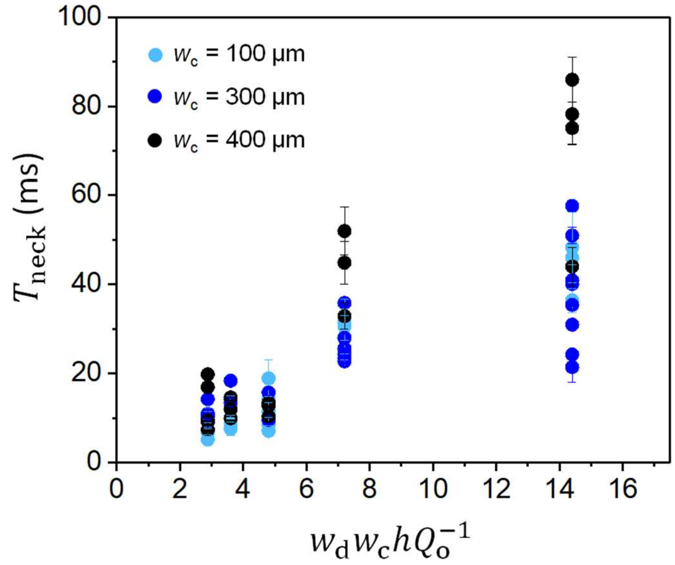

Supplementary Fig. 10 Plot of necking time, T_{neck}, of droplet breakup versus $w_d w_c h Q_o^{-1}$. Each dot corresponds to one experimental condition of w_d (width of branch), w_c (width of main channel), h (channel height), and Q_o (flow rate of oil), with the error bars indicating the standard deviations from measurements of at least 500 droplets.

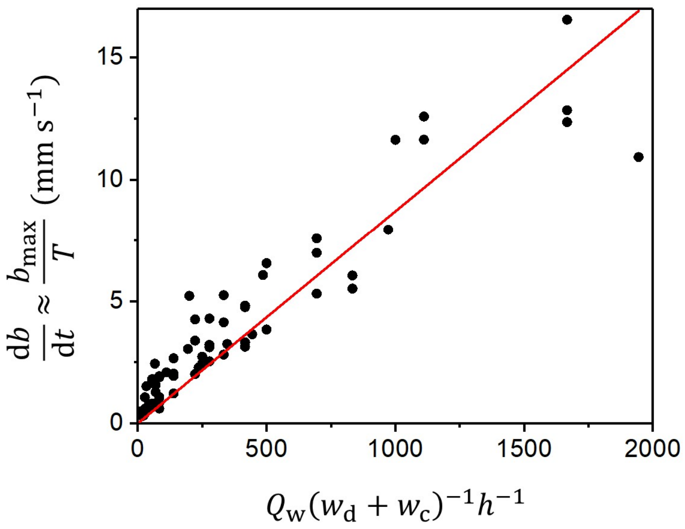

Supplementary Fig. 11 Plot of the protrusion rate, $\frac{\mathrm{d}b}{\mathrm{d}t}$ versus $Q_w(w_d+w_c)^{-1}h^{-1}$. Each dot corresponds to the average value of the linear slopes of *b*(*t*) (the maximum protrusion length, b_{max} over a period of droplet breakup, *T*) from independent experimental conditions, with Q_w (flow rate of water), w_d (width of branch), w_c (width of main channel), and *h* (channel height).

Supplementary Note 1

Derivation of Equation (4)

The net force acting on the dispersed phase at the end of filling stage is 0, as calculated according to

$$F_\tau + F_\mathrm{R} + F_\gamma = \frac{\mu_\mathrm{o} Q_\mathrm{o} b}{w_g h} + \frac{12\mu_\mathrm{o} Q_\mathrm{o} b^2}{{w_g}^2 h} - \gamma h = 0 \qquad (1)$$

For two interfaces to retain the in-phase synchronization states, i.e., $b_2(t) = b_1(t)$, the gap width $w_\mathrm{g} = w_\mathrm{c} - (b_1(t) + b_2(t)) = w_\mathrm{c} - 2b_1(t)$ should remain positive. Because we assume that $b_1(t)$ changes linearly with respect to time, i.e., $\frac{\mathrm{d}b_1(t)}{\mathrm{d}t}$ is constant, we substitute $b = B \cdot T_\mathrm{fill}$, and $w_\mathrm{g} = w_\mathrm{c} - 2B \cdot T_\mathrm{fill}$ to Supplementary Equation 1, where B is assumed to be a constant $\frac{\mathrm{d}b_1(t)}{\mathrm{d}t}$ for simplicity. Now, we can solve for T_fill, the time when the net force becomes zero:

$$T_\mathrm{fill} = \frac{w_\mathrm{c}}{B} \frac{\frac{4h}{w_\mathrm{c}} + \mathrm{Ca} - \sqrt{\mathrm{Ca}}\sqrt{\frac{48h}{w_\mathrm{c}} + \mathrm{Ca}}}{\frac{8h}{w_\mathrm{c}} - 20\mathrm{Ca}} \qquad (2)$$

Then, the right-hand side of Equation (6) in the main text, $0.5w_\mathrm{c} - \frac{\mathrm{d}b_1(t)}{\mathrm{d}t} \cdot T_\mathrm{fill}$ simplifies to $\frac{6w_\mathrm{c}}{11 + \sqrt{1 + \frac{48h}{w_\mathrm{c}\ \mathrm{Ca}}}}$. The Equation (6) can be rewritten as $\frac{w_\mathrm{d} w_\mathrm{c}}{w_\mathrm{d} + w_\mathrm{c}} \cdot \frac{Q_\mathrm{w}}{Q_\mathrm{o}} = A \cdot \frac{6w_\mathrm{c}}{11\ \ \sqrt{1 + \frac{48h}{w_\mathrm{c}\ \mathrm{Ca}}}}$, which simplifies to Equation (7) in the main text.

Supplementary Note 2

Discussion of *α* in the model of out-of-phase mode

In the representative calculation for the out-of-phase state shown in Fig. 5c, the model converges to the out-of-phase state with a fixed α. We found that with the correction factor $\xi = 0$, the breakup of one dispersed phase occurs at the very beginning of the necking stage of the other dispersed phase. However, the experimentally observed state of out-of-phase synchronization exhibits a time delay between the start of the necking stage in one branch and the breakup in the other branch. Thus, the role of ξ and, ultimately, the force balance in the drop breakup of the out-of-phase state needs additional investigation. Furthermore, the approximation of the linear increase in $b_i(t)$ is not applicable in many cases in the out-of-phase mode. For instance, $\frac{\mathrm{d}b}{\mathrm{d}t}$ (Fig. 5b in the main text and Supplementary Fig. 9b) tends to change when entering the necking stage. Finally, the lag stage should be taken into account to predict α. Although we neglected the lag stage by incorporating it into the filling stage, the interface can retract considerably into the branch after the breakup, especially

when Q_w is small (Supplementary Fig. 9a); when $\frac{Q_w w_c}{Q_o w_d} < 0.03$, $b(t)$ usually remains negative in the lag stage before the filling stage begins, for a time comparable to the filling or necking duration. However, for a sufficiently large value of Q_w, as shown in Figs. 1, 3, and 5 in the main text, considering only the filling and necking stage was adequate for describing the breakup process.
