# Supplementary material for: Phase synchronization of fluid-fluid interfaces as hydrodynamically coupled oscillators
Source: Nat Commun. 2020 Oct 15;11:5221. doi: 10.1038/s41467-020-18930-7 (PMC7562928; doi:10.1038/s41467-020-18930-7)
Supplement: Supplementary file 3 — Description of Additional Supplementary Files [file 41467_2020_18930_MOESM3_ESM.pdf]

Description of Additional Supplementary Files

File name: Supplementary Movie 1
Description: Out-of-phase synchronization of droplet breakup

File name: Supplementary Movie 2
Description: Transition between out-of-phase and in-phase synchronization of droplet breakup.

File name: Supplementary Movie 3
Description: In-phase synchronization of droplet breakup.

File name: Supplementary Movie 4
Description: Recovery of the in-phase synchronization after flow perturbation.

File name: Supplementary Movie 5
Description: Droplet flow maintaining the side by side configuration at different regions in the main channel towards the outlet, after the synchronized droplet breakup in-phase
